# Redetermined structure of gossypol (*P*3 polymorph)

**DOI:** 10.1107/S205698901500941X

**Published:** 2015-06-03

**Authors:** Muhabbat Honkeldieva, Rishad Kunafiev, Hayrullo I. Hamidov

**Affiliations:** a Institute of Biorganic Chemistry, Mirzo-Ulughbek Str. 83, Tashkent 100125, Uzbekistan

**Keywords:** crystal structure, redetermination, gossypol, polymorph, hydrogen bonding

## Abstract

An improved crystal structure of the title compound, C_30_H_30_O_8_ (systematic name: 1,1′,6,6′,7,7′-hexa­hydroxy-5,5′-diisopropyl-3,3′-dimeth­yl[2,2′-bi­naphthalene]-8,8′-dicarbaldehyde), was determined based on modern CCD data. Compared to the previous structure [Talipov *et al.* (1985). *Khim. Prirod. Soedin. (Chem. Nat. Prod.)*, **6**, 20–24], geometrical precision has been improved (typical C—C bond-distance s.u. = 0.002 Å in the present structure compared to 0.005 Å in the previous structure) and the locations of several H atoms have been corrected. The gossypol mol­ecules are in the aldehyde tautomeric form and the dihedral angle between the naphthyl fragments is 80.42 (4)°. Four intra­molecular O—H⋯O hydrogen bonds are formed. In the crystal, inversion dimers with graph-set motif *R*_2_^2^(20) are formed by pairs of O—H⋯O hydrogen bonds; another pair of O—H⋯O hydrogen bonds with the same graph-set motif links the dimers into [001] chains. The packing of such chains in the crystal leads to the formation of channels (diameter = 5–8 Å) propagating in the [101] direction. The channels presumably contain highly disordered solvent mol­ecules; their contribution to the scattering was removed with the SQUEEZE [Spek (2015). *Acta Cryst*. C**71**, 9–18] routine in *PLATON* and the stated mol­ecular mass, density *etc.*, do not take them into account.

## Related literature   

For the previous structure determination of gossypol *P*3 polymorph, see: Talipov *et al.*, (1985 ▸). For details of the extraction and synthesis of gossypol and its derivatives, see: Adams *et al.* (1960 ▸). For its synthesis and biological activities, see: Baram & Ismailov (1993 ▸); Polsky *et al.* (1989 ▸); Radloff *et al.* (1985 ▸). For information on crystalline inclusion compounds, see: Ibragimov & Talipov (1999 ▸, 2004 ▸); Ibragimov *et al.* (1997 ▸); Gdaniec *et al.* (1996 ▸); Talipov *et al.* (1988 ▸). For the use of SQUEEZE, see: Spek (2015 ▸).
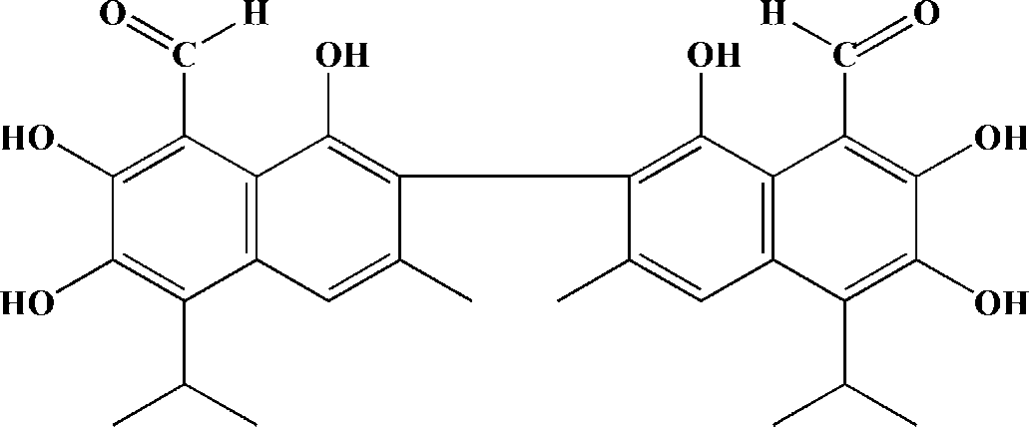


## Experimental   

### Crystal data  

C_30_H_30_O_8_
*M* *_r_* = 518.54
Monoclinic, 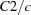

*a* = 21.2196 (4) Å
*b* = 19.0886 (2) Å
*c* = 15.2564 (2) Å
β = 113.262 (2)°
*V* = 5677.29 (16) Å^3^
*Z* = 8
Cu *K*α radiation
μ = 0.73 mm^−1^
*T* = 293 K
0.30 × 0.30 × 0.30 mm

### Data collection  

Oxford Diffraction Xcalibur Ruby diffractometer
Absorption correction: multi-scan (*CrysAlis PRO*; Oxford Diffraction, 2009 ▸) *T*_min_ = 0.730, *T*_max_ = 1.000
13408 measured reflections
5810 independent reflections
4382 reflections with *I* > 2σ(*I*)
*R*_int_ = 0.021

### Refinement  

*R*[*F* ^2^ > 2σ(*F* ^2^)] = 0.047
*wR*(*F* ^2^) = 0.161
*S* = 1.11
5810 reflections
374 parameters
H atoms treated by a mixture of independent and constrained refinement
Δρ_max_ = 0.44 e Å^−3^
Δρ_min_ = −0.30 e Å^−3^

### 

Data collection: *CrysAlis PRO* (Oxford Diffraction, 2009 ▸); cell refinement: *CrysAlis PRO*; data reduction: *CrysAlis PRO*; program(s) used to solve structure: *SHELXS97* (Sheldrick, 2008 ▸); program(s) used to refine structure: *SHELXL2014* (Sheldrick, 2015 ▸); molecular graphics: *OLEX2* (Dolomanov *et al.*, 2009 ▸); software used to prepare material for publication: *OLEX2*.

## Supplementary Material

Crystal structure: contains datablock(s) I. DOI: 10.1107/S205698901500941X/hb7412sup1.cif

Structure factors: contains datablock(s) I. DOI: 10.1107/S205698901500941X/hb7412Isup2.hkl

Supporting information file. DOI: 10.1107/S205698901500941X/hb7412Isup3.cml

**Fig. 1.**
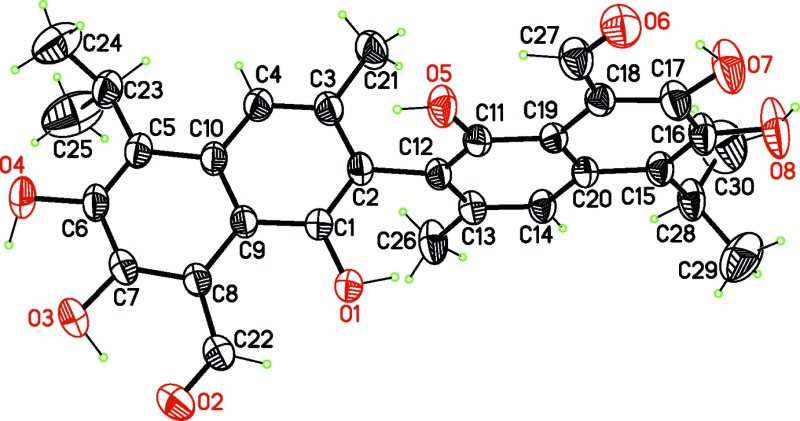
The molecular structure of title compound, with displacement ellipsoids shown at the 50% probability level.

. DOI: 10.1107/S205698901500941X/hb7412fig1.tif
The mol­ecular structure of title compound, with displacement ellipsoids shown at the 50% probability level.

**Fig. 2.**
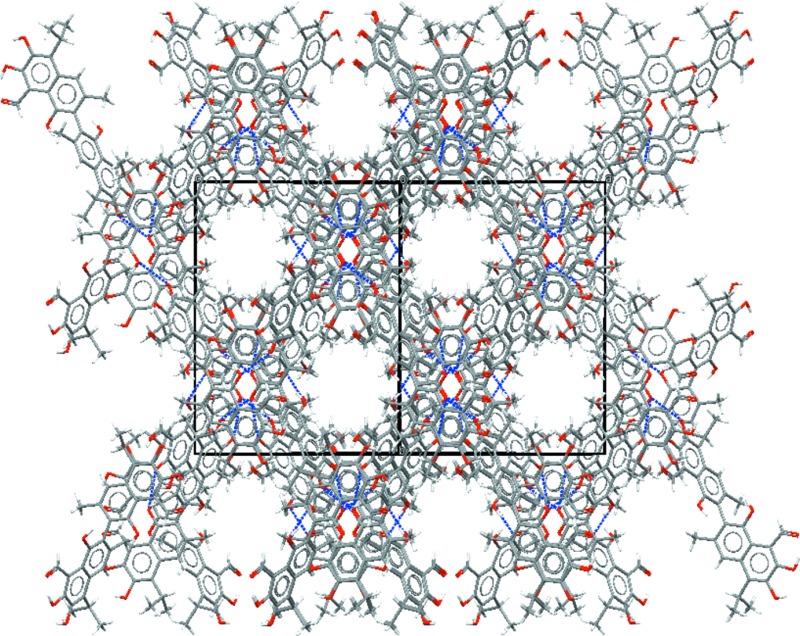
A packing diagram for title compound.

. DOI: 10.1107/S205698901500941X/hb7412fig2.tif
A packing diagram for title compound.

CCDC reference: 1401388

Additional supporting information:  crystallographic information; 3D view; checkCIF report

## Figures and Tables

**Table 1:** Hydrogen-bond geometry (, )

*D*H*A*	*D*H	H*A*	*D* *A*	*D*H*A*
O1H1O6^i^	0.90(2)	2.16(2)	2.9692(17)	150(2)
O3H3O2	0.90(3)	1.59(3)	2.454(2)	160(3)
O5H5O3^ii^	0.83(2)	2.30(2)	2.9546(17)	136(2)
O4H4*A*O3	0.98(4)	1.88(4)	2.601(2)	128(3)
O4H4*A*O5^ii^	0.98(4)	2.46(4)	3.278(2)	141(3)
O7H7O6	0.92(3)	1.63(3)	2.479(2)	152(3)
O8H8O7	0.87(4)	2.02(4)	2.575(2)	120(3)
C22H22O1	0.93	2.12	2.721(2)	121
C26H26*B*O8^iii^	0.96	2.55	3.483(2)	165
C27H27O4^iv^	0.93	2.31	3.138(2)	148
C27H27O5	0.93	2.07	2.727(2)	127

Symmetry codes: (i) 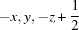; (ii) 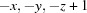; (iii) 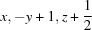; (iv) 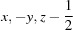.

## supplementary crystallographic information

### S1.1. Synthesis and crystallization

Preparative details of the material

### S1.2. Refinement

Crystal data, data collection and structure refinement details are summarized in Table 1.

### S2. Results and discussion

Comment

Gossypol, a phenolic pigment extracted from cotton seeds [Adams *et al.*, 1960], demonstrates a wide range of biological activity [Baram *et al.*, 1993; Polsky *et al.*, 1989: Radloff *et al.*,1985] and versatile host properties [Ibragimov, Talipov. 1999; 2004; Gdaniec *et al.*, 1996]. Unique ability as a host compound to form crystalline inclusion compounds with many organic solvents makes gossypol an inter­esting object of solid supra­molecular chemistry. Gossypol has also been found to form pseudopolymorphic structures with same guest molecule, e.g., clathrates formed with di­chloro­methane [Ibragimov *et al.*, 1997] and di­ethyl ether [Talipov *et al.*, 1988]. Unsolvated polymorphs of the compound are also known [Gdaniec *et al.*, 1996]. In the crystal of the title compound, gossypol (1,1',6,6',7,7'-hexa­hydroxy -5,5'diiso­propyl - 3,3'di­methyl­[2,2' - bi­naphthalene] - 8,8'- dicarboxaldehyde), C_30_H_30_O_8_, is one independent molecule in the asymmetric part of the unit cell. The crystals of the title compound were obtained after decomposition of gossypol clathrate with di­chloro­methane, where the single crystals are not destroyed and their cell volumes are only reduced by ~4%. In the title compound gossypol molecules are in the aldehyde tautomeric form (Fig. 1). H-bonds O4—H···O3 (O8—H···O7) and O3—H···O2 (O7—H···O6) form five- and six-membered rings. Naphthyl fragments C(1)—C(10) (C7 0.07A) and C(11)—C(20) (C12 0.04 A) are planar and dihedral angle between their planes are equal to 80.42 (4)°. One of the most commonly found associations is a centrosymmetric dimer that is linked by two pairs O5–H···O3 and O4–H···O5 hydrogen bonds and hydro­phobic stacking inter­actions between two of the naphthalene rings [Gdaniec *et al.*, 1996]. In the title crystal centrosymmetric dimers are formed as above, these assemble into extended serpentine chains by other pair of hydrogen bonds O1—H···O6 directed along the c axis through a twofold rotation axis with direction [0 1 0]. The packing of such chains in the crystal leads to the formation of broadly rough channels (Fig. 2) (where diameter varied 5-7 A), parallel to the *ac* diagonal.

### Crystal data

**Table:**

C_30_H_30_O_8_	*D*_x_ = 1.213 Mg m^−^^3^
*M**_r_* = 518.54	Melting point: 455 K
Monoclinic, *C*2/*c*	Cu *K*α radiation, λ = 1.54184 Å
*a* = 21.2196 (4) Å	Cell parameters from 6170 reflections
*b* = 19.0886 (2) Å	θ = 3.9–75.6°
*c* = 15.2564 (2) Å	µ = 0.73 mm^−^^1^
β = 113.262 (2)°	*T* = 293 K
*V* = 5677.29 (16) Å^3^	Prism, light brown
*Z* = 8	0.30 × 0.30 × 0.30 mm
*F*(000) = 2192	

### Data collection

**Table:**

Oxford Diffraction Xcalibur Ruby diffractometer	5810 independent reflections
Radiation source: fine-focus sealed tube	4382 reflections with *I* > 2σ(*I*)
Graphite monochromator	*R*_int_ = 0.021
Detector resolution: 10.2576 pixels mm^-1^	θ_max_ = 75.8°, θ_min_ = 3.9°
ω scans	*h* = −26→25
Absorption correction: multi-scan (*CrysAlis PRO*; Oxford Diffraction, 2009)	*k* = −23→20
*T*_min_ = 0.730, *T*_max_ = 1.000	*l* = −17→19
13408 measured reflections	

### Refinement

**Table:**

Refinement on *F*^2^	Hydrogen site location: mixed
Least-squares matrix: full	H atoms treated by a mixture of independent and constrained refinement
*R*[*F*^2^ > 2σ(*F*^2^)] = 0.047	*w* = 1/[σ^2^(*F*_o_^2^) + (0.0994*P*)^2^ + 0.2158*P*] where *P* = (*F*_o_^2^ + 2*F*_c_^2^)/3
*wR*(*F*^2^) = 0.161	(Δ/σ)_max_ < 0.001
*S* = 1.11	Δρ_max_ = 0.44 e Å^−^^3^
5810 reflections	Δρ_min_ = −0.30 e Å^−^^3^
374 parameters	Extinction correction: *SHELXL2014* (Sheldrick, 2008), Fc^*^=kFc[1+0.001xFc^2^λ^3^/sin(2θ)]^-1/4^
0 restraints	Extinction coefficient: 0.00026 (5)
Primary atom site location: structure-invariant direct methods	

### Special details

**Table:**

Experimental. Absorption correction: CrysAlisPro, Oxford Diffraction Ltd., Version 1.171.33.40 (release 27-04-2009 CrysAlis171 .NET) (compiled Apr 27 2009,10:20:11) Empirical absorption correction using spherical harmonics, implemented in SCALE3 ABSPACK scaling algorithm.
Geometry. All e.s.d.'s (except the e.s.d. in the dihedral angle between two l.s. planes) are estimated using the full covariance matrix. The cell e.s.d.'s are taken into account individually in the estimation of e.s.d.'s in distances, angles and torsion angles; correlations between e.s.d.'s in cell parameters are only used when they are defined by crystal symmetry. An approximate (isotropic) treatment of cell e.s.d.'s is used for estimating e.s.d.'s involving l.s. planes.

### Fractional atomic coordinates and isotropic or equivalent isotropic displacement parameters (Å^2^)

**Table:**

	*x*	*y*	*z*	*U*_iso_*/*U*_eq_	
C1	0.04702 (8)	0.15341 (8)	0.55658 (11)	0.0429 (3)	
C2	0.10359 (8)	0.18397 (8)	0.54801 (11)	0.0447 (3)	
C3	0.16553 (8)	0.14655 (9)	0.57686 (13)	0.0547 (4)	
C4	0.16876 (9)	0.08045 (9)	0.61308 (14)	0.0564 (4)	
H4	0.2100	0.0562	0.6322	0.068*	
C5	0.11810 (9)	−0.02370 (8)	0.65817 (12)	0.0518 (4)	
C6	0.06006 (9)	−0.05514 (8)	0.65630 (12)	0.0512 (4)	
C7	−0.00297 (9)	−0.01904 (8)	0.62878 (11)	0.0480 (4)	
C8	−0.00888 (8)	0.05136 (8)	0.60352 (11)	0.0465 (3)	
C9	0.04970 (8)	0.08525 (8)	0.59537 (11)	0.0433 (3)	
C10	0.11255 (8)	0.04756 (8)	0.62278 (12)	0.0472 (4)	
C11	0.09182 (8)	0.26474 (7)	0.41457 (11)	0.0428 (3)	
C12	0.09891 (8)	0.25641 (7)	0.50805 (11)	0.0427 (3)	
C13	0.10483 (9)	0.31582 (8)	0.56537 (11)	0.0477 (4)	
C14	0.10549 (9)	0.38096 (8)	0.52707 (11)	0.0466 (4)	
H14	0.1090	0.4202	0.5649	0.056*	
C15	0.10583 (9)	0.46057 (7)	0.39810 (11)	0.0479 (4)	
C16	0.10033 (11)	0.46668 (8)	0.30665 (13)	0.0605 (5)	
C17	0.08923 (10)	0.40867 (9)	0.24522 (12)	0.0559 (4)	
C18	0.08645 (8)	0.34087 (7)	0.27596 (11)	0.0434 (3)	
C19	0.09259 (7)	0.33132 (7)	0.37324 (10)	0.0393 (3)	
C20	0.10103 (7)	0.39099 (7)	0.43325 (10)	0.0407 (3)	
C21	0.22811 (10)	0.17856 (12)	0.5684 (2)	0.0817 (7)	
H21A	0.2176	0.1905	0.5030	0.123*	
H21B	0.2652	0.1455	0.5899	0.123*	
H21C	0.2413	0.2201	0.6070	0.123*	
C22	−0.07150 (10)	0.08521 (11)	0.59498 (18)	0.0725 (6)	
H22	−0.0747	0.1336	0.5872	0.087*	
C23	0.18663 (11)	−0.06196 (10)	0.69912 (17)	0.0703 (6)	
H23	0.2214	−0.0300	0.6947	0.084*	
C24	0.18936 (15)	−0.12839 (16)	0.6460 (2)	0.1056 (9)	
H24A	0.1578	−0.1621	0.6520	0.158*	
H24B	0.2350	−0.1473	0.6725	0.158*	
H24C	0.1771	−0.1178	0.5798	0.158*	
C25	0.20640 (17)	−0.0777 (2)	0.8058 (2)	0.1318 (14)	
H25A	0.2067	−0.0348	0.8390	0.198*	
H25B	0.2512	−0.0986	0.8320	0.198*	
H25C	0.1736	−0.1094	0.8128	0.198*	
C26	0.11096 (13)	0.30777 (10)	0.66665 (13)	0.0706 (6)	
H26A	0.0693	0.2881	0.6666	0.106*	
H26B	0.1188	0.3528	0.6971	0.106*	
H26C	0.1487	0.2772	0.7007	0.106*	
C27	0.07713 (11)	0.28657 (9)	0.20641 (13)	0.0591 (4)	
H27	0.0776	0.2404	0.2260	0.071*	
C28	0.11809 (12)	0.52550 (8)	0.46049 (13)	0.0629 (5)	
H28	0.1217	0.5097	0.5234	0.075*	
C29	0.05884 (17)	0.57648 (13)	0.4239 (2)	0.1027 (9)	
H29A	0.0533	0.5928	0.3618	0.154*	
H29B	0.0680	0.6156	0.4668	0.154*	
H29C	0.0176	0.5534	0.4199	0.154*	
C30	0.18621 (17)	0.56118 (16)	0.4751 (2)	0.1138 (11)	
H30A	0.2213	0.5263	0.4878	0.171*	
H30B	0.1987	0.5929	0.5281	0.171*	
H30C	0.1813	0.5867	0.4185	0.171*	
O1	−0.01395 (6)	0.18820 (6)	0.52761 (9)	0.0547 (3)	
O2	−0.12161 (8)	0.05299 (9)	0.59743 (15)	0.0931 (6)	
O3	−0.05495 (7)	−0.05604 (7)	0.63411 (10)	0.0615 (3)	
O4	0.06055 (9)	−0.12336 (6)	0.68412 (11)	0.0692 (4)	
O5	0.08350 (7)	0.20689 (6)	0.35806 (9)	0.0590 (3)	
O6	0.06858 (8)	0.29704 (7)	0.12265 (9)	0.0644 (4)	
O7	0.08315 (11)	0.42438 (8)	0.15667 (10)	0.0847 (5)	
O8	0.10566 (13)	0.53046 (7)	0.26920 (13)	0.1047 (8)	
H1	−0.0135 (11)	0.2226 (13)	0.4879 (17)	0.077 (7)*	
H3	−0.0864 (15)	−0.0220 (15)	0.6236 (19)	0.095 (9)*	
H5	0.0875 (12)	0.1717 (13)	0.3922 (16)	0.075 (7)*	
H7	0.0769 (15)	0.3820 (18)	0.126 (2)	0.111 (10)*	
H4A	0.0132 (19)	−0.1253 (18)	0.680 (3)	0.147 (13)*	
H8	0.094 (2)	0.523 (2)	0.209 (3)	0.150 (14)*	

### Atomic displacement parameters (Å^2^)

**Table:**

	*U*^11^	*U*^22^	*U*^33^	*U*^12^	*U*^13^	*U*^23^
C1	0.0461 (8)	0.0365 (7)	0.0473 (7)	0.0015 (6)	0.0197 (6)	0.0053 (6)
C2	0.0508 (8)	0.0338 (7)	0.0500 (8)	−0.0038 (6)	0.0206 (7)	0.0057 (6)
C3	0.0477 (9)	0.0472 (8)	0.0699 (10)	−0.0027 (7)	0.0240 (8)	0.0120 (8)
C4	0.0468 (9)	0.0466 (9)	0.0758 (11)	0.0064 (7)	0.0242 (8)	0.0159 (8)
C5	0.0574 (9)	0.0379 (8)	0.0594 (9)	0.0047 (7)	0.0224 (8)	0.0097 (7)
C6	0.0695 (10)	0.0341 (7)	0.0532 (9)	−0.0007 (7)	0.0275 (8)	0.0091 (6)
C7	0.0574 (9)	0.0423 (8)	0.0479 (8)	−0.0098 (7)	0.0246 (7)	0.0035 (6)
C8	0.0485 (8)	0.0411 (7)	0.0512 (8)	−0.0023 (6)	0.0213 (7)	0.0078 (6)
C9	0.0474 (8)	0.0357 (7)	0.0476 (7)	−0.0029 (6)	0.0198 (6)	0.0047 (6)
C10	0.0502 (8)	0.0361 (7)	0.0543 (8)	0.0005 (6)	0.0197 (7)	0.0085 (6)
C11	0.0479 (8)	0.0323 (7)	0.0509 (8)	−0.0046 (6)	0.0224 (6)	−0.0012 (6)
C12	0.0448 (8)	0.0337 (7)	0.0506 (8)	−0.0049 (6)	0.0199 (6)	0.0045 (6)
C13	0.0583 (9)	0.0410 (8)	0.0445 (8)	−0.0044 (7)	0.0209 (7)	0.0028 (6)
C14	0.0616 (9)	0.0335 (7)	0.0466 (8)	−0.0037 (6)	0.0233 (7)	−0.0017 (6)
C15	0.0642 (10)	0.0327 (7)	0.0498 (8)	−0.0031 (6)	0.0256 (7)	0.0002 (6)
C16	0.0982 (14)	0.0334 (7)	0.0564 (9)	−0.0045 (8)	0.0377 (10)	0.0046 (7)
C17	0.0837 (12)	0.0442 (8)	0.0472 (8)	−0.0039 (8)	0.0336 (8)	0.0027 (7)
C18	0.0487 (8)	0.0366 (7)	0.0474 (8)	−0.0038 (6)	0.0217 (6)	−0.0010 (6)
C19	0.0396 (7)	0.0347 (7)	0.0449 (7)	−0.0022 (5)	0.0179 (6)	0.0016 (6)
C20	0.0445 (7)	0.0328 (6)	0.0453 (7)	−0.0029 (5)	0.0184 (6)	0.0018 (6)
C21	0.0505 (10)	0.0701 (13)	0.1269 (19)	−0.0021 (9)	0.0375 (11)	0.0316 (13)
C22	0.0609 (11)	0.0571 (10)	0.1109 (17)	0.0042 (9)	0.0459 (11)	0.0266 (11)
C23	0.0620 (11)	0.0503 (10)	0.0965 (15)	0.0120 (8)	0.0291 (10)	0.0236 (10)
C24	0.0982 (19)	0.0973 (19)	0.123 (2)	0.0388 (16)	0.0454 (17)	0.0012 (17)
C25	0.097 (2)	0.178 (3)	0.0897 (19)	0.064 (2)	0.0040 (15)	0.005 (2)
C26	0.1167 (17)	0.0480 (9)	0.0512 (9)	−0.0095 (10)	0.0375 (10)	0.0026 (8)
C27	0.0861 (13)	0.0436 (8)	0.0529 (9)	−0.0074 (8)	0.0332 (9)	−0.0031 (7)
C28	0.1036 (15)	0.0339 (7)	0.0555 (9)	−0.0079 (8)	0.0361 (10)	−0.0011 (7)
C29	0.146 (3)	0.0668 (14)	0.0910 (17)	0.0310 (15)	0.0415 (17)	−0.0126 (12)
C30	0.132 (3)	0.0823 (17)	0.120 (2)	−0.0452 (17)	0.0423 (19)	−0.0307 (16)
O1	0.0512 (6)	0.0445 (6)	0.0724 (8)	0.0076 (5)	0.0288 (6)	0.0194 (5)
O2	0.0632 (9)	0.0838 (11)	0.1484 (16)	0.0039 (7)	0.0589 (10)	0.0385 (10)
O3	0.0680 (8)	0.0479 (7)	0.0764 (8)	−0.0133 (6)	0.0369 (7)	0.0076 (6)
O4	0.0928 (10)	0.0367 (6)	0.0868 (10)	0.0017 (6)	0.0449 (8)	0.0167 (6)
O5	0.0923 (9)	0.0322 (5)	0.0590 (7)	−0.0084 (6)	0.0367 (6)	−0.0027 (5)
O6	0.0897 (9)	0.0578 (7)	0.0516 (7)	−0.0050 (6)	0.0343 (6)	−0.0086 (5)
O7	0.1637 (17)	0.0497 (7)	0.0551 (8)	−0.0093 (9)	0.0587 (9)	0.0028 (6)
O8	0.222 (2)	0.0387 (7)	0.0723 (10)	−0.0190 (10)	0.0786 (13)	0.0043 (6)

### Geometric parameters (Å, º)

**Table:**

C1—C2	1.387 (2)	C19—C20	1.428 (2)
C1—C9	1.421 (2)	C21—H21A	0.9600
C1—O1	1.3633 (18)	C21—H21B	0.9600
C2—C3	1.405 (2)	C21—H21C	0.9600
C2—C12	1.4985 (19)	C22—H22	0.9300
C3—C4	1.368 (2)	C22—O2	1.242 (2)
C3—C21	1.513 (2)	C23—H23	0.9800
C4—H4	0.9300	C23—C24	1.519 (4)
C4—C10	1.406 (2)	C23—C25	1.541 (4)
C5—C6	1.360 (2)	C24—H24A	0.9600
C5—C10	1.451 (2)	C24—H24B	0.9600
C5—C23	1.523 (2)	C24—H24C	0.9600
C6—C7	1.413 (2)	C25—H25A	0.9600
C6—O4	1.3685 (18)	C25—H25B	0.9600
C7—C8	1.390 (2)	C25—H25C	0.9600
C7—O3	1.3392 (19)	C26—H26A	0.9600
C8—C9	1.450 (2)	C26—H26B	0.9600
C8—C22	1.436 (2)	C26—H26C	0.9600
C9—C10	1.425 (2)	C27—H27	0.9300
C11—C12	1.383 (2)	C27—O6	1.234 (2)
C11—C19	1.4218 (19)	C28—H28	0.9800
C11—O5	1.3688 (18)	C28—C29	1.512 (3)
C12—C13	1.407 (2)	C28—C30	1.533 (4)
C13—C14	1.376 (2)	C29—H29A	0.9600
C13—C26	1.507 (2)	C29—H29B	0.9600
C14—H14	0.9300	C29—H29C	0.9600
C14—C20	1.410 (2)	C30—H30A	0.9600
C15—C16	1.358 (2)	C30—H30B	0.9600
C15—C20	1.4509 (19)	C30—H30C	0.9600
C15—C28	1.521 (2)	O1—H1	0.90 (2)
C16—C17	1.409 (2)	O3—H3	0.90 (3)
C16—O8	1.368 (2)	O4—H4A	0.98 (4)
C17—C18	1.386 (2)	O5—H5	0.83 (2)
C17—O7	1.339 (2)	O7—H7	0.92 (3)
C18—C19	1.449 (2)	O8—H8	0.87 (4)
C18—C27	1.440 (2)		
			
C2—C1—C9	122.05 (14)	C3—C21—H21A	109.5
O1—C1—C2	120.70 (13)	C3—C21—H21B	109.5
O1—C1—C9	117.25 (13)	C3—C21—H21C	109.5
C1—C2—C3	119.61 (14)	H21A—C21—H21B	109.5
C1—C2—C12	120.39 (14)	H21A—C21—H21C	109.5
C3—C2—C12	120.00 (13)	H21B—C21—H21C	109.5
C2—C3—C21	120.78 (15)	C8—C22—H22	118.4
C4—C3—C2	119.13 (15)	O2—C22—C8	123.15 (18)
C4—C3—C21	120.08 (16)	O2—C22—H22	118.4
C3—C4—H4	118.5	C5—C23—H23	107.2
C3—C4—C10	123.02 (15)	C5—C23—C25	110.12 (19)
C10—C4—H4	118.5	C24—C23—C5	114.39 (19)
C6—C5—C10	117.78 (15)	C24—C23—H23	107.2
C6—C5—C23	120.46 (15)	C24—C23—C25	110.5 (2)
C10—C5—C23	121.73 (16)	C25—C23—H23	107.2
C5—C6—C7	122.28 (14)	C23—C24—H24A	109.5
C5—C6—O4	121.16 (16)	C23—C24—H24B	109.5
O4—C6—C7	116.53 (15)	C23—C24—H24C	109.5
C8—C7—C6	121.68 (14)	H24A—C24—H24B	109.5
O3—C7—C6	115.50 (14)	H24A—C24—H24C	109.5
O3—C7—C8	122.71 (16)	H24B—C24—H24C	109.5
C7—C8—C9	117.94 (14)	C23—C25—H25A	109.5
C7—C8—C22	116.05 (14)	C23—C25—H25B	109.5
C22—C8—C9	125.83 (14)	C23—C25—H25C	109.5
C1—C9—C8	123.34 (14)	H25A—C25—H25B	109.5
C1—C9—C10	117.61 (13)	H25A—C25—H25C	109.5
C10—C9—C8	118.97 (13)	H25B—C25—H25C	109.5
C4—C10—C5	120.68 (15)	C13—C26—H26A	109.5
C4—C10—C9	118.55 (13)	C13—C26—H26B	109.5
C9—C10—C5	120.76 (14)	C13—C26—H26C	109.5
C12—C11—C19	122.99 (13)	H26A—C26—H26B	109.5
O5—C11—C12	119.42 (13)	H26A—C26—H26C	109.5
O5—C11—C19	117.59 (13)	H26B—C26—H26C	109.5
C11—C12—C2	119.23 (13)	C18—C27—H27	117.7
C11—C12—C13	119.66 (13)	O6—C27—C18	124.58 (16)
C13—C12—C2	121.04 (14)	O6—C27—H27	117.7
C12—C13—C26	120.37 (14)	C15—C28—H28	106.9
C14—C13—C12	118.54 (14)	C15—C28—C30	111.80 (19)
C14—C13—C26	121.09 (15)	C29—C28—C15	112.44 (18)
C13—C14—H14	118.5	C29—C28—H28	106.9
C13—C14—C20	123.09 (14)	C29—C28—C30	111.5 (2)
C20—C14—H14	118.5	C30—C28—H28	106.9
C16—C15—C20	117.91 (14)	C28—C29—H29A	109.5
C16—C15—C28	119.79 (14)	C28—C29—H29B	109.5
C20—C15—C28	122.29 (14)	C28—C29—H29C	109.5
C15—C16—C17	122.66 (14)	H29A—C29—H29B	109.5
C15—C16—O8	121.16 (15)	H29A—C29—H29C	109.5
O8—C16—C17	116.18 (15)	H29B—C29—H29C	109.5
C18—C17—C16	121.81 (14)	C28—C30—H30A	109.5
O7—C17—C16	114.77 (15)	C28—C30—H30B	109.5
O7—C17—C18	123.41 (15)	C28—C30—H30C	109.5
C17—C18—C19	117.74 (13)	H30A—C30—H30B	109.5
C17—C18—C27	115.76 (14)	H30A—C30—H30C	109.5
C27—C18—C19	126.49 (14)	H30B—C30—H30C	109.5
C11—C19—C18	123.67 (13)	C1—O1—H1	108.3 (15)
C11—C19—C20	116.70 (13)	C7—O3—H3	100.3 (18)
C20—C19—C18	119.62 (12)	C6—O4—H4A	98 (2)
C14—C20—C15	120.88 (13)	C11—O5—H5	107.5 (16)
C14—C20—C19	118.94 (12)	C17—O7—H7	104.4 (19)
C19—C20—C15	120.17 (13)	C16—O8—H8	105 (3)

### Hydrogen-bond geometry (Å, º)

**Table:**

*D*—H···*A*	*D*—H	H···*A*	*D*···*A*	*D*—H···*A*
O1—H1···O6^i^	0.90 (2)	2.16 (2)	2.9692 (17)	150 (2)
O3—H3···O2	0.90 (3)	1.59 (3)	2.454 (2)	160 (3)
O5—H5···O3^ii^	0.83 (2)	2.30 (2)	2.9546 (17)	136 (2)
O4—H4*A*···O3	0.98 (4)	1.88 (4)	2.601 (2)	128 (3)
O4—H4*A*···O5^ii^	0.98 (4)	2.46 (4)	3.278 (2)	141 (3)
O7—H7···O6	0.92 (3)	1.63 (3)	2.479 (2)	152 (3)
O8—H8···O7	0.87 (4)	2.02 (4)	2.575 (2)	120 (3)
C22—H22···O1	0.93	2.12	2.721 (2)	121
C26—H26*B*···O8^iii^	0.96	2.55	3.483 (2)	165
C27—H27···O4^iv^	0.93	2.31	3.138 (2)	148
C27—H27···O5	0.93	2.07	2.727 (2)	127

Symmetry codes: (i) −*x*, *y*, −*z*+1/2; (ii) −*x*, −*y*, −*z*+1; (iii) *x*, −*y*+1, *z*+1/2; (iv) *x*, −*y*, *z*−1/2.

## References

[bb1] Adams, R., Geissman, T. A. & Edwards, J. D. (1960). *Chem. Rev.* **60**, 555–574. 10.1021/cr60208a002 13681414

[bb2] Baram, N. I. & Ismailov, A. I. (1993). *Khim. Prir. Soedin.* p. 334.

[bb4] Dolomanov, O. V., Bourhis, L. J., Gildea, R. J., Howard, J. A. K. & Puschmann, H. (2009). *J. Appl. Cryst.* **42**, 339–341.

[bb5] Gdaniec, M., Ibragimov, B. T. & Talipov, S. A. (1996). *Gossypol*, edited by D. D. MacNicol, F. Toda & R. Bishop, *Solid-state supramolecular chemistry: crystal engineering*, Vol. 6, *Comprehensive supramolecular chemistry*, pp. 117–146. Oxford: Pergamon Press.

[bb6] Ibragimov, B. T. & Talipov, S. A. (1999). *J. Struct. Chem.* **40**, 686–704.

[bb7] Ibragimov, B. T. & Talipov, S. A. (2004). *Gossypol*, in *Encyclopedia of Supramolecular Chemistry*, edited by J. L. Atwood & J. W. Steed, pp. 606–614. New York: Dekker.

[bb8] Ibragimov, B. T., Tiljakov, Z. G., Beketov, K. M. & Talipov, S. A. (1997). *J. Inclusion Phenom. Mol. Recognit. Chem.* **27**, 99–104.

[bb9] Oxford Diffraction (2009). *CrysAlis PRO*. Oxford Diffraction Ltd, Yarnton, England.

[bb10] Polsky, B., Segal, S. J., Baron, P. A., Gold, J. W. M., Ueno, H. & Armstrong, D. (1989). *Contraception*, **39**, 579–587. 10.1016/0010-7824(89)90034-6 2473865

[bb11] Radloff, R. I., Deck, L. M., Royer, R. E. & Vander Jagt, D. L. (1985). *Pharmacol. Res. Commun.* **18**, 1063. 10.1016/0031-6989(86)90023-8 3025895

[bb12] Sheldrick, G. M. (2008). *Acta Cryst.* A**64**, 112–122. 10.1107/S0108767307043930 18156677

[bb13] Sheldrick, G. M. (2015). *Acta Cryst.* C**71**, 3–8.

[bb14] Spek, A. L. (2015). *Acta Cryst.* C**71**, 9–18. 10.1107/S2053229614024929 25567569

[bb15] Talipov, S. A., Ibragimov, B. T., Nazarov, G. B., Aripov, T. F. & Sadikov, A. S. (1985). *Khim. Prir. Soedin (Russ.) (Chem. Nat. Compd.)*, pp. 835–836.

[bb16] Talipov, S. A., Ibragimov, B. T., Tishchenko, G. N., Aripov, T. F., Nazarov, G. B., Strokopytov, B. V. & Polyakov, K. M. (1988). *Kristallografiya*, **33**, 384–389.

