# Supplementary figures and images for: Redetermined structure of gossypol (P3 polymorph)
Source: Acta Crystallogr E Crystallogr Commun. 2015 Jun 3;71(Pt 7):o442–3. doi: 10.1107/S205698901500941X (PMC4518924; doi:10.1107/S205698901500941X)

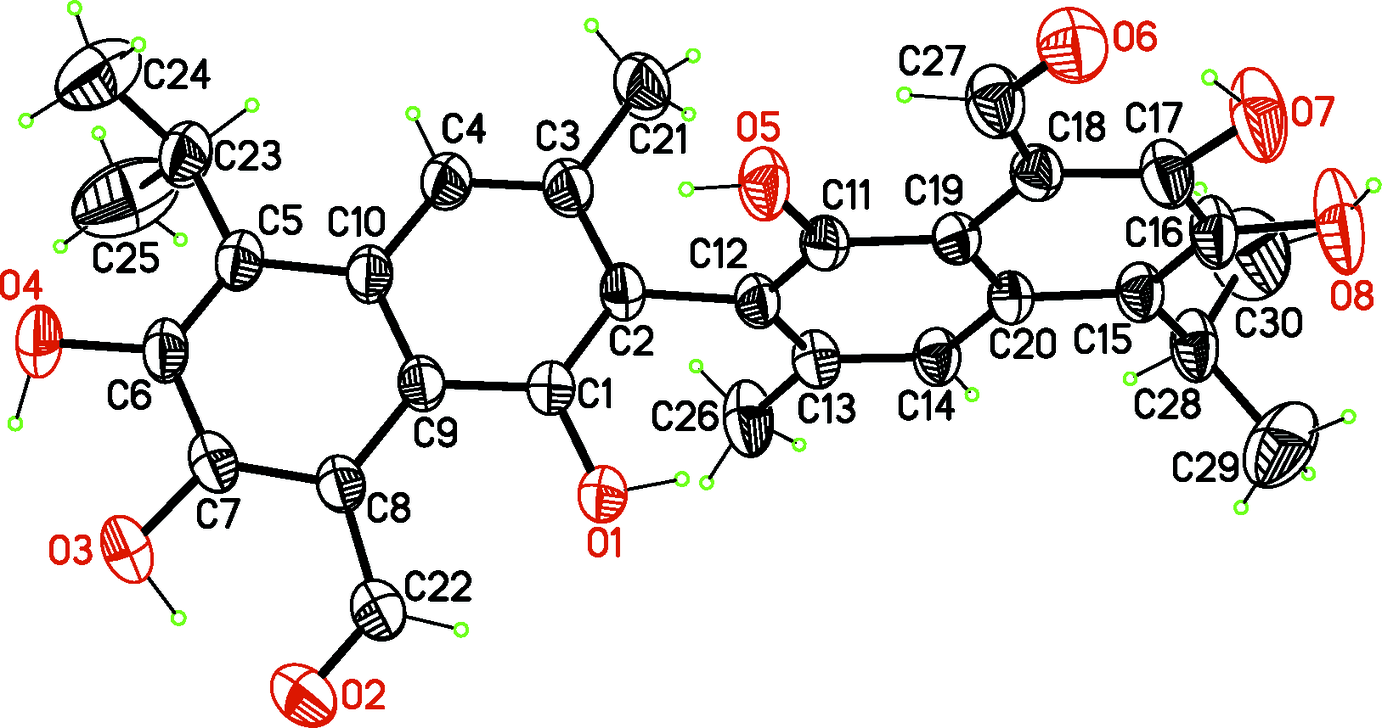

Supplement: Supplementary file 4 [file e-71-0o442-fig1.tif]

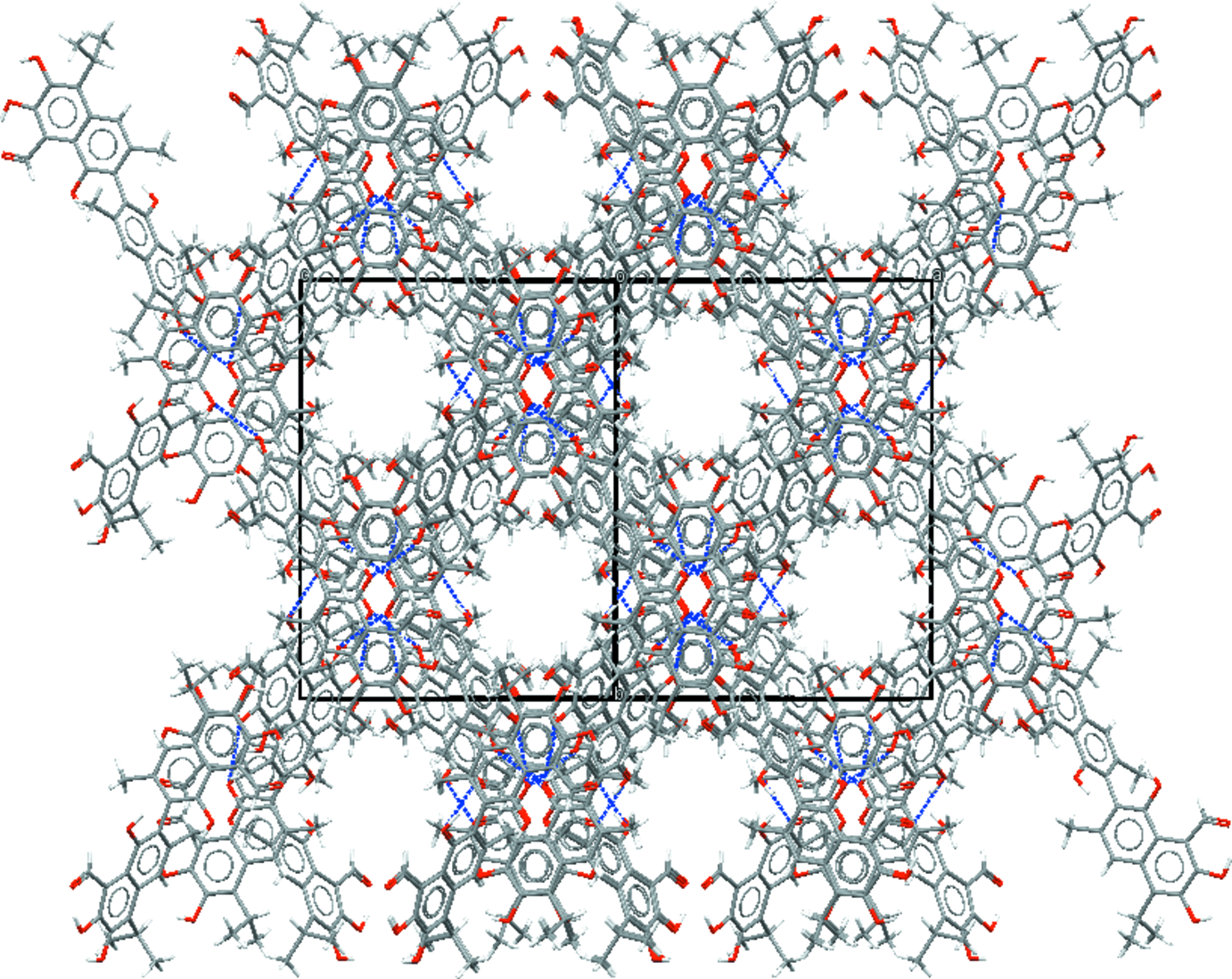

Supplement: Supplementary file 5 [file e-71-0o442-fig2.tif]
